# The Role of pH Fronts in Reversible Electroporation

**DOI:** 10.1371/journal.pone.0017303

**Published:** 2011-04-29

**Authors:** Pablo Turjanski, Nahuel Olaiz, Felipe Maglietti, Sebastian Michinski, Cecilia Suárez, Fernando Victor Molina, Guillermo Marshall

**Affiliations:** 1 Laboratorio de Sistemas Complejos, Departamento de Computacion, Facultad de Ciencias Exactas y Naturales, Universidad de Buenos Aires, Buenos Aires, Argentina; 2 Consejo Nacional de Investigaciones Cienticas y Tecnicas, Buenos Aires, Argentina; 3 INQUIMAE, Facultad de Ciencias Exactas y Naturales, Universidad de Buenos Aires, Buenos Aires, Argentina; University of California at Berkeley, United States of America

## Abstract

We present experimental measurements and theoretical predictions of ion transport in agar gels during reversible electroporation (ECT) for conditions typical to many clinical studies found in the literature, revealing the presence of pH fronts emerging from both electrodes. These results suggest that pH fronts are immediate and substantial. Since they might give rise to tissue necrosis, an unwanted condition in clinical applications of ECT as well as in irreversible electroporation (IRE) and in electrogenetherapy (EGT), it is important to quantify their extent and evolution. Here, a tracking technique is used to follow the space-time evolution of these pH fronts. It is found that they scale in time as 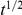, characteristic of a predominantly diffusive process. Comparing ECT pH fronts with those arising in electrotherapy (EChT), another treatment applying constant electric fields whose main goal is tissue necrosis, a striking result is observed: anodic acidification is larger in ECT than in EChT, suggesting that tissue necrosis could also be greater. Ways to minimize these adverse effects in ECT are suggested.

## Introduction

During the last decade, pulsed electric fields were explored in local tumor treatment based upon electroporation, a technique in which pulsed electric fields are employed to disturb cell membrane integrity creating pores across it. Among them, electrochemotherapy (ECT), irreversible electroporation (IRE) and electrogenetherapy (EGT). ECT combines a reversible electroporation (cell-membrane permeabilizing electric pulses below the irreversible threshold) with non-permeant or poorly-permeant anticancer drugs to potentiate their entry to the cell thus their intrinsic citotoxicity [1], [2]. Since its beginnings in the late 1980s, ECT has evolved into a clinically verified palliative or cytoreductive treatment for cutaneous and subcutaneous tumor nodules of different malignancies in Europe and the United States. Typical ECT treatment in humans consists in a train of 8 square pulses of high electric field (around 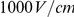) and very short duration (around 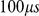) delivered at 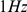 [3].

A recent derivation of ECT is EGT (introduction of plasmids or oligonucleotides to the cell by electropermeabilization [4]). This new technique is being intensely studied due to its potential as a nonviral gene-delivery system. EGT has even found to be effective in neoplastic clearance and complete protection against mammary carcinoma development in transgenic mice [5]. This opens the possibility that a combination of ECT and EGT achieve both local and systemic control of heretofore incurable cancers [6].

Another recent derivation of ECT is IRE, introduced in [7], an irreversible electroporation (electric pulses above the irreversible threshold), without thermal effects, that leaves intact main tissue structures [8]. An important difference of IRE relative to ECT is that the killing of cells is induced by permanent membrane disruption without any drug or DNA delivery. Whether ECT, EGT or IRE, al these techniques have some undesired side effects (loss of cell viability, uncontrolled necrosis, plasmid damage) that is necessary to minimize. An excellent recent review of electroporation techniques in biology and medicine can be found in [9].

Previous ECT modeling approaches in the literature include [10], [11] that compute electric field distributions based on 2D models and show that the applied voltage, configuration of the electrodes and electrode position need to be chosen specifically for each individual case. [12] calculates transmembrane potential (TMP) and electroporation density across membrane of spheroidal cells subject to ultrashort, high-intensity pulses, showing that the TMP induced by pulsed external voltages can be substantially higher in oblate spheroidal as compared to spherical or prolate spheroidal cells. [13] introduces a model describing the creation and resealing of pores at a single whole cell. [14] simulates the effects of external electric fields on clusters of excitable cells, showing that the stimulation of a given cell depends in part on the arrangement of cells within the field and not simply the location within the field. [15] computes the electric field distribution in deep-seated tumors taking into account that tissue conductivity changes during the delivery of electric pulses. [16] caculates the mass transfer into cells during ECT introducing a multiscale model that couples an external electrical field model at tissue level, an electroporation-driven mass transfer model at a single cell level, and a macroscopic mass transfer diffusion model in tissue. Typical IRE applications in models consists in electric fields around 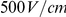, using around 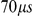 delivered at 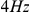.

In this paper we look into the electroporation process from a new angle apparently overseen in the literature, the role of pH in ECT modeling based on ion transport and associated pH changes that take place during the treatment. This analysis is developed through in vitro gel measurements and theoretical modeling drawing from previous experience in the electrochemical treatment of tumors (EChT), another electrochemical-based antitumoral treatment that applies direct currents with the aim of eliminating tumors mainly by necrosis (see [17]–[22]) and in electrochemical deposition in thin layer cells [23]. It is well known that, during EChT, two opposing pH fronts emerge from both electrodes (acid from anode and basic from cathode) until collision somewhere between them. These pH alterations can be used to predict the extent of the tumor necrotic area [24] which may be, in part, attributed to electrocoagulation [22].

While in EChT tumor necrosis is the main goal of the treatment, and in IRE it may contribute to tumor destruction, in ECT and EGT it is usually avoided because of its collateral effects. A common problem of ECT and EGT is their low cell viability and, in relation to EGT, its low transfection efficiency compared with other transfection methods [25]. It has been suggested that these effects may be strongly dependent on the change of pH induced by electrolysis during the process. Significant pH alterations of the medium during EGT may have deleterious effects over the plasmids used for the delivery, as DNA denaturation is prominently affected by pH [26], [27].

The plan of the paper follows: the second section, materials and methods, presents a description of the experimental procedures and of the in silico modeling, and the third section describes main results and a discussion of them , and finally, some general conclusions.

## Materials and Methods

**Figure 1:**
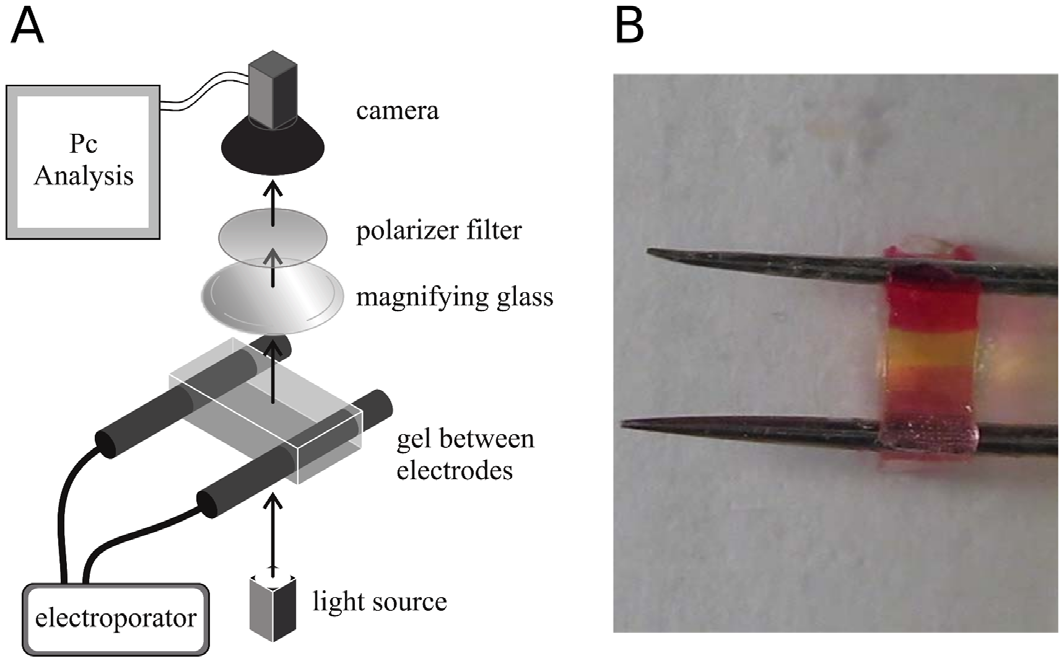
Experimental setup. a) The electroporator is conected to the electrodes placed in the gel. Images of the process during the ECT are collected through an optic system by a videocamera and then analyzed by specific software. b) Detail of the electrodes placed in the gel revealing the existence of acid and basic pH fronts.

Experimental in vitro modeling is based in the application of ECT at different pulse amplitudes and duration to a thin film gel system. The experimental setup is shown in figure 1A. It consists in a thin 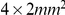 film, 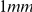 thick, 1% agar-agar gel with NaCl at physiological concentration (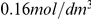), 1% methyl red (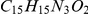, point transition pH 6.2), 1% phenolphtalein (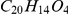, point transition pH 8.3) and pH 7. Two platinum rod electrodes 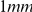 in diameter were placed at the ends of the gel, 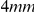 from each other. Figure 1B shows the electrode and gel configuration with anodic and cathodic pH fronts in pink and red colors, respectively. Eight electric pulses of 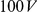, 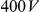 or 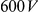 (corresponding to 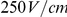, 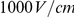 and 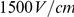) and 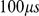, 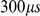 or 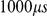 were delivered at 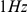 by an square wave electroporator (ECM 830, BTX-Harvard Apparatus, USA). Visual front tracking of pH indicator color changes emerging from both electrodes was obtained using magnifying lenses. Video camera images (Powershot SX20 IS) were captured at 30 fps with a resolution of 312 pixels/cm, processed and analyzed at 10 fps with the ImageJ graphic package [28]. All experiments were conducted at room temperature with no significant changes observed during the process.

In silico modelling is based in the description of the ECT process by a 1D system of partial differential equations governing ion transport and the electric field in a four component electrolyte and its numerical solution by deterministic finite difference methods. Assuming that ion transport is governed by diffusion and migration and electroneutrality holds true, the in silico ECT 1D model can be approximated by a new split model describing ion transport in a two-step procedure. During the ON-time step, electric current is present and transport is governed by migration and diffusion, during the OFF-time step electric current is absent and transport is solely governed by diffusion. This splitting model allows a multitime step in which the ON time step is several orders of magnitude smaller than the OFF time step.

During the ON-time step, the in silico ECT model is described by the Nernst-Planck equations for the concentration of ions in a four component electrolyte under potentiostatic conditions. The model includes five unknown variables: proton, hydroxide, sodium and chloride concentrations, and the electric field. The equations are written as:
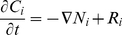
(1)


Here, the molar flux is: 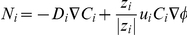. where 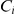, 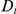, 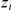 and 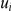 are the concentration, diffusion coefficient, charge number and mobility of the species 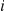, respectively (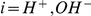 and 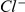). 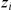 are signed quantities, being positive for cations and negative for anions. 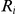 represents the production of the species 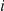 through chemical reactions in the electrolyte. 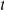 is the time and 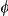 is the electric field. Details about boundary conditions and the way the system of partial differential equations is solved can be found in [21].

During the OFF-time step, the in silico ECT model is similar to the one corresponding to the ON-time step but now the molar flux becomes:
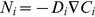
(2)


Regarding boundary conditions, mass transport of species 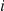 across both electrode surfaces is assumed non-existent. Then, for 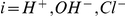 and 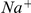:
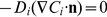
(3)


The split system of partial differential equations is solved, successively in time, in a two-step procedure (ON-time and OFF-time steps), in a fixed domain on a two-dimensional space-time uniform grid using strongly implicit finite differences (details can be found in [29]). During the ON-time step, the time step is diminished several orders of magnitude to account for the microsecond migration time scale, yielding a remarkable robust numerical algorithm. The computational model was written in the C++ language and implemented on a Intel(R) Core(TM) i5 class computer under Ubuntu Linux OS. The nonlinear equation resulting from the approximation of the boundary conditions is solved by the Newton's method, using Multidimensional Root-Finding routines from the GNU Scientific Library (GSL).

Simulation starts with the ON-time step system. At 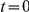, there are no concentration gradients throughout the electrolyte. Table 1 presents the input parameters for the computational model. Initial salt concentration is set to be 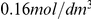, which is close to that found in plasma and interstitial fluids [30]. Initial pH is set to be 7 (neutral). Published diffusion coefficients of ions in liquid medium [31] were adequately reduced to describe diffusion in a gel medium. These new values are in the range of published experimental data for sodium chloride diffusion in gels [32]. During the OFF-time step the system is solved using as initial conditions the solution obtained from the ON-time step.

**Table 1:** Input parameters of the mathematical model.

Parameter	Value	Ref	Parameter	Value	Ref
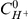	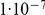 mol/dm 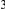		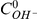	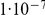 mol/dm 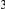	
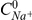	0.16 mol/dm 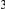	[30]	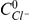	0.16 mol/dm 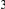	[30]
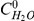	55.5 mol/dm 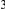		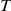	298 K	
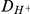	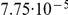 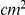 /s		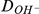	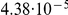 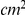 /s	
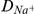	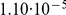 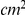 /s		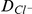	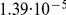 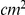 /s	
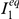	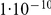 A/ 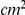	[34]	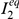	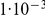 A/ 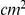	[35]
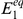	0.816 V	[31]	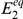	1.407 V	[31]
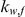	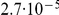 s 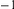	[36]	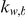	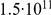 dm 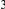 /(mol s)	[36]

## Results and Discussion

**Figure 2:**
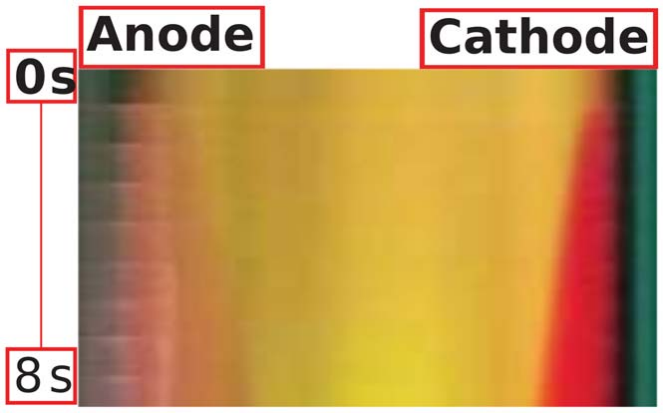
Evolution of the anodic acid (pink) and cathodic basic (red) pH fronts. Space-time measurement of pH variation in an in vitro gel under ECT (8 pulses, 400 V, 300 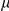
s, 1 Hz).

Figure 2 presents the space-time representation of an ECT gel experiment showing the evolution of the acid and basic pH distribution. Acid and basic pH fronts are represented by pink and red pixels, respectively. This figure is constructed from a stack of pH spatial distributions for different times, and unveils the existence of significant pH gradients during a typical ECT. It is readily seen the larger extension of the anodic pH front relative to the cathodic one, though both pH indicators change color at the same distance from neutrality (6.2 and 8.3 for acid and basic indicators respectively). During the final writing of this paper we learned that in [25], a significant pH alteration of the medium due to ECT was observed too, though with a different experimental model.

**Figure 3:**
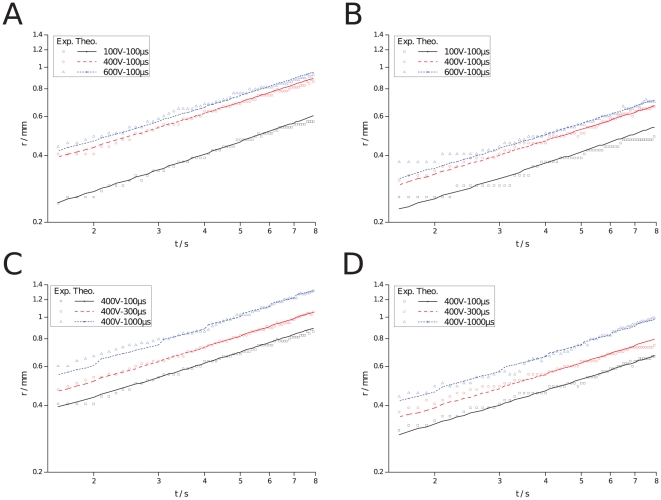
pH front tracking during an ECT, measured (symbols) and predicted (lines), in semi-log scale. a) Anodic front, pulse length of 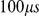, 8 pulses, 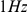 and pulse amplitudes of 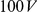, 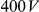 and 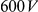. b) Cathodic front, same parameters as in a). c) Anodic front, pulse amplitude of 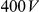, 8 pulses, 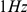 and pulse lengths of 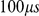, 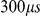 and 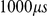. d) Cathodic front, same parameters as in c).

Figure 3 shows experimental results (symbols) and theoretical predictions (lines) of pH front tracking, in semi-log scale, during ECT for different pulse lengths and amplitudes. Figures 3A and 3B presents anodic and cathodic fronts, respectively, for a pulse length of 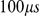 (8 pulses, 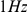) and pulse amplitudes of 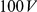, 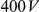 and 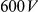. Figures 3C and 3D presents anodic and cathodic fronts, respectively, for a pulse amplitude of 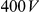 (8 pulses, 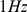) and pulse lengths of 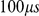, 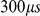 and 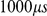. A comparison of theoretical predictions of pH front tracking with experimental measurements shows a good agreement. Increasing pulse length or amplitude results in a higher pH front speed, hence, the possibility of a larger necrotic area. Here too, acid pH fronts are faster than basic ones, as previously mentioned. This probably is due to the larger diffusion and migration coefficients of protons. Nevertheless, in all cases, curve slopes are about 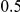 (calculation not shown) which means that pH fronts scale in time as 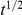, characteristic of a predominantly diffusion-controlled process. This is because the total ON-time during the whole process is much shorter (several orders of magnitude) than the OFF-time.

**Figure 4:**
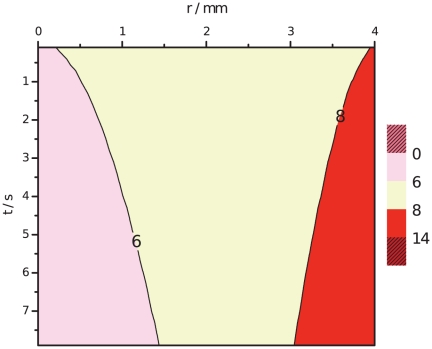
Evolution of the anodic acid (pink) and cathodic basic (red) pH fronts. Space-time prediction of pH variation under ECT (anodic and cathodic contour lines at pH 6 and 8, respectively), at 8 pulses, 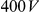, 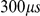, 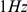.

Figure 4 presents the predicted space and time representation of an ECT, for the same conditions as in the experiment of figure 2, showing the evolution of the acid and basic pH fronts, represented by pink and red pixels, respectively. The figure again reveals the existence of significant pH gradients during a typical ECT. A comparison with the experimental measurements in figure 2 shows a remarkable agreement. In both, it is observed the larger extension of the anodic pH front relative to the cathodic one.

**Figure 5:**
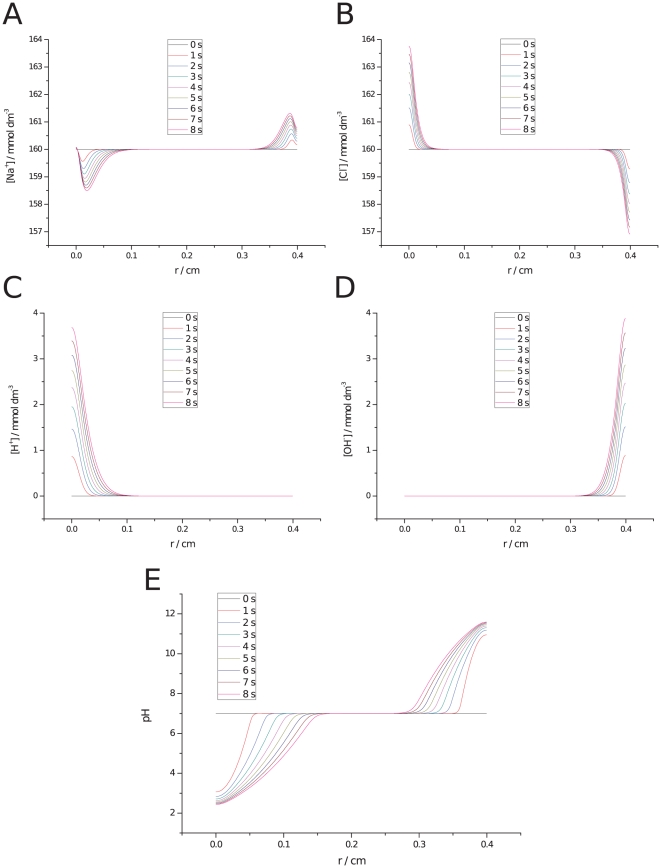
Predicted ionic concentration and pH variation during ECT. Ionic concentrations (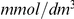) and pH distribution in space (cm), (8 pulses of 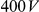 and 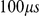 at 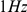) at different times (0–8 s). Anode at left and cathode at right: a) Sodium, b) Chlorine, c) Protons, d) Hydroxides, and e) pH distribution.

Figure 5 shows theoretical predictions of the spatial concentration of the four ionic species and the pH distribution for an ECT treatment (train of 8 pulses at 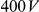, 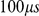 and 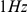). It can be observed an increase in chlorine and a decrease in sodium concentrations at the anode while the opposite occurs at the cathode (figures 5A and 5B). The extreme anodic acidification and cathodic basification induced during ECT can be traced to proton and hydroxide increments at anode and cathode, respectively (figures 5C and 5D). Here again, it is observed the large anodic acid and cathodic basic fronts expanding in time towards each other (figure 5E).

**Figure 6:**
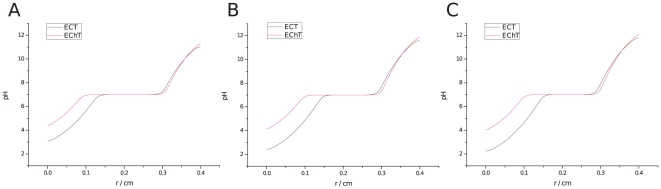
Predicted pH variation during ECT (black) and EChT(red), with same electric current dosage. Anode at left and cathode at right: a) 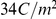, ECT: 8 pulses of 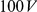 and 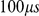 at 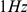, 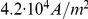 and 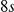 total, EChT: 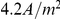, 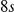; b) 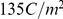, ECT (8 pulses of 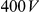 and 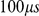 at 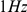, 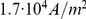 and 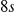 total), EChT (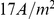, 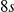); c) 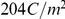, ECT (8 pulses of 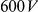 and 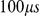 at 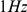, 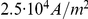 and 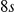 total), EChT (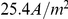, 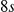).

Figure 6 shows the predicted spatial distribution of pH during ECT and EChT in black and red lines, respectively, for the same electric current dosage. Comparing both fronts, a striking result is observed: anodic acidification is larger in ECT than in EChT, suggesting that tissue necrosis could also be greater.

The previous theoretical result was experimentally corroborated by a pH indicator strip (Merk, range 0,5–5) placed on the electrodes and wetted with a NaCl solution at physiological concentration. Upon ECT pulse delivery (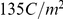, 8 pulses, 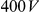, 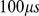, 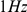), the strip zone located at the anode changed color (from blue, basal state, to yellow) corresponding to a pH value of around 2 according to the color table. The same procedure, but now applying EChT at the same dosage (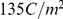, 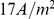, 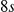), turned the strip zone into orange, corresponding to a pH value of around 4. For a better assessment of pH values, images were taken with a Casio FH-25 High Speed Camera and analyzed using Image J. Histograms of the color of the strips and of the color table were made, revealing that the ECT strip turned into pH 2.5 while the EChT strip turned into pH 4 (results not shown).

Note that current applied, for the pulse length tested, is four orders of magnitude higher for ECT than for EChT. This implies larger electrochemical reactions and, locally and instantaneously, larger ECT anodic proton generation. This could be the main reason of the greater acidity achieved by ECT. This may be relevant for ECT treatment optimization, where it is desired to apply an effective dosage while minimizing pH effects that can lead to necrosis and plasmid damage. One way to achieve this could be by minimizing voltages and pulse number while maximizing pulse lengths as far as possible. In fact, this new low-current, low-voltage and long-duration pulse procedure, has been proved recently to be safer and more efficient in DNA electrodelivery [4], [33].

In summary, in this work we studied the electroporation process from a new angle apparently overseen in the literature, the role of ion transport and associated pH changes in ECT modeling. This analysis was developed through in vitro gel measurements and theoretical modeling. We presented experimental evidence that ion transport in in vitro gels during reversible electroporation, for conditions typical to many clinical studies found in the literature, unveil the presence of pH fronts emerging from both electrodes. These pH fronts are immediate and substantial. Using a tracking technique to follow the space-time evolution of this fronts, we found that they scale in time as 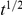, characteristic of a predominantly diffusive process. This information is extremely useful for predicting tissue treatment extent. Moreover, we introduced a new splitting theoretical model describing ion transport in ECT in a two-step procedure, the first step or ON-time step describing ion transport governed by migration and diffusion, and the second step or OFF-time step describing ion transport governed solely by diffusion. This splitting allows a multitime step in which the ON-time step is several orders of magnitude smaller than the OFF time step, yielding a remarkable robust numerical algorithm. Theoretical pH front tracking has an excellent correlation with experimental measurements. Moreover, its predictions of the comparison of ECT pH fronts with those arising in EChT revealed a striking result that was experimentally corroborated: anodic acidification is larger in ECT than in EChT, suggesting that tissue necrosis could also be greater. The quantification of pH extension and evolution is relevant for optimizing ECT treatment, where it is desired to apply an effective dosage while minimizing pH alterations leading to necrosis and plasmid damage. One way to achieve this could be designing protocols minimizing voltage and pulse number while maximizing pulse lengths as far as possible. It is expected that the results of this work might help in optimizing electroporation-based tumor therapies through minimizationof pH adverse effects.
